# Impact of Antioxidant-Enriched Edible Gel Coatings and Bio-Based Packaging on Cherry Tomato Preservation

**DOI:** 10.3390/gels10090549

**Published:** 2024-08-24

**Authors:** Corinne Giacondino, Alessandra De Bruno, Davide Puntorieri, Martina Pizzimenti, Amalia Piscopo

**Affiliations:** 1 Department AGRARIA, University Mediterranea of Reggio Calabria, 89124 Reggio Calabria, Italy; corinne.giacondino@unirc.it (C.G.); puntorieridavide@gmail.com (D.P.); martina.pizzimenti@outlook.it (M.P.); 2 Department of Human Sciences and Promotion of the Quality of Life, San Raffaele University, 00166 Rome, Italy; alessandra.debruno@uniroma5.it

**Keywords:** antioxidant extract, bio-based packaging, cherry tomatoes, edible gel coating, food preservation, lemon pomace, shelf life extension

## Abstract

This research investigates the effects of using edible gel coatings and bio-based packaging materials on extending the shelf life of cherry tomatoes. Two edible gel coatings (guar gum and guar gum +5% of a lemon (*Citrus limon* (L.) Osbeck pomace extract obtained in the research laboratory) were applied on cherry tomatoes, then they were packaged in bio-based materials (cellulose tray + PLA lid). Guar gum, glycerol, sorbitol, extra virgin olive oil, and tween 20 were used in coating formulation. Uncoated tomatoes packed in bio-based materials and conventional plastic (PET trays + lid) were tested as a control. Samples were stored for 45 days at 20 °C and their quality parameters were evaluated. Coated tomatoes maintained firmness and weight, and the enriched coated samples showed a significant increase in phenol content, derived from the antioxidant extract. Samples packed in PET showed a sensory unacceptability (<4.5) after 45 days correlated with a greater decline in firmness (from 10.51 to 5.96 N) and weight loss (from 7.06 to 11.02%). Therefore, edible gel coating and bio-based packaging proved to be effective in maintaining the overall quality of cherry tomatoes for 45 days, offering a promising approach to reduce plastic polymer use and food waste.

## 1. Introduction

Nowadays food industry is looking for solutions that can combine maintaining product quality and freshness and, thus, reducing postharvest losses and extending product shelf life [1]. In this context, food packaging plays a key role, and, in fact, research pushes toward the use of compostable biodegradable packaging materials to replace conventional plastic polymers, widely used for packaging fresh products, but strongly involved in environmental pollution [2]. Currently, biopolymers used for biodegradable packaging include polylactic acid (PLA), a thermoplastic aliphatic polyester, synthesized by the fermentation of sugary raw materials, such as corn. PLA is used in food packaging for short-lived products which could replace traditional plastics such as polyethylene (LDPE, HDPE), polystyrene (PS), and, polyethylene terephthalate (PET), due to its various properties (good workability, transparency, high disintegration in compost, appearance, high mechanical strength, low toxicity) [3]. Another abundant and used polymer in the food packaging industry is cellulose, derived from the cell wall of plants, which is appreciated for its biocompatibility and environmental sustainability [4]. 

Among sustainable and innovative alternatives, the application of edible gel coatings to fruit and vegetables is widely known. They are commonly based on natural polymers, such as polysaccharides, proteins, lipids, or their combination, combined with plasticizers, emulsifiers, and other bioactive components for the creation of a protective barrier capable of slowing gas transmission, moisture loss, and preserving the appearance of the product throughout the storage period [5,6,7]. Among polysaccharides, guar gum, a galactomannan derived from the endosperm of a legume plant (*Cyamopsis tetragonoloba*) [1], is widely used for fruit quality preservation [1,8,9], due to its ability to form films, non-toxicity, biocompatibility, and biodegradability [10]. However, it has some limitations related to poor mechanical properties and high hydrophilicity [11]. Usually, for the coating of fresh produce, polysaccharides are combined with lipids to improve mechanical strength and water barrier properties to optimize moisture retention within the fresh product [5]. Numerous studies have been conducted by a combination of guar gum with other polymers and additives to obtain better performance of composite gel coatings [12,13,14]. 

Tomato (*Solanum lycopersicum* L.) fruit is on the basis of the Mediterranean diet, and is among the most widely consumed food products and also serves as a staple food for many nations [15]. Tomatoes are a rich source of nutrients and health-beneficial compounds but have a limited postharvest shelf life. Their climacteric nature, in fact, makes them susceptible to rapid deterioration caused by biochemical and physiological processes (e.g., senescence, ethylene synthesis, transpiration) that result in nutrient loss and economic damage [16,17]. Controlling the postharvest life of cherry tomatoes is therefore an important focus of research to improve quality and prolong storage. 

Among the potentially suitable methods to control the shelf life of cherry tomatoes, bioplastic materials packaging is an interesting sustainable option, as reported in the recent literature [2,18,19] with the successful use of PLA compared with PET. Furthermore, the use of composite edible gel coatings proved to be effective in maintaining the quality characteristics of tomatoes during the storage period [20,21,22,23]. In particular, Kumar et al. [21] reported the application of a composite coating formulated from whey protein isolate, xanthan gum, clove oil, and glycerol monostearate on tomato, showing effectiveness on shelf life extension and sustaining quality attributes of tomatoes during 15 days of storage at 20 °C. A composite coating of gum arabic (GA) and carboxymethylcellulose (CMC) was applied to tomatoes showing promising results, compared to the single coating, in prolonging the ripening phase, delaying senescence, and increasing the acceptability of tomato fruits for 20 days [22]. Due to the positive results of the application of composite coatings on tomatoes for 20 days of shelf life, the study aims to investigate the application of a new formulation, not yet considered in the literature, in the further extension of shelf life up to 45 days.

Moreover, the functional properties of an edible film can also be improved through the incorporation of natural additives from plant extracts. They could act as carriers of bioactive compounds that can contribute to maintaining quality attributes during storage and further prolong the shelf life [11]. Several films with polysaccharides functionalized with natural extracts have been applied to tomatoes [20,24]. The application of plant extracts could guarantee the replacement of synthetic additives, which are increasingly less appreciated by the consumer [5]. Bioactive compounds added to coatings could derive from the recovery of agri-food by-products as a sustainable way to enhance and reduce food waste and, also, limit environmental impact [25]. Some studies reported the addition, of different coatings, of food by-product extracts as natural additives to provide bioactive compounds in tomato fruits like pomegranate peel extract [21,26] or a lemon pomace extract added to a pectin coating and applied to fresh-cut carrots with excellent results on structural integrity, reduced microbial activity, and higher levels of bioactive compounds and antioxidant activity [27]. In this regard, in the present study, the extract from lemon (*Citrus limon* (L.) Osbeck) pomace, consisting of various solid residues (seeds, peel, pulp) was used and considered a potential burden for the environment. Retaining important components, such as phenolic compounds [28], its addition in a coating formulation for cherry tomatoes could improve their functional proprieties and shelf life.

The objective of this study was to evaluate the effects of using biodegradable packaging (cellulose tray + PLA lid) in comparison with plastic packaging (PET trays and lid), actually widely used on the market, on the shelf life of cherry tomatoes. Moreover, the efficiency of two different composite gel coatings was evaluated in maintaining the quality characteristics of tomatoes during a 45-day storage period at 20 °C. The addition to gel coating formulation of a lemon pomace extract, rich in phenolic compounds, was particularly tested for providing bioactive compounds and extending cherry tomato shelf life. This approach could contribute to extending the shelf life of the fruit and enhance the use of a by-product extract with a green approach. 

## 2. Results and Discussion

### 2.1. Characterization Analysis of the Film

The resulting films were thin, homogeneous, and flexible, were easily removed from the petri dishes, and had an average thickness of 0.1 ± 0.01 mm. The films produced were sufficiently thin to qualify as films as defined by The American Society for Testing and Materials (ASTM) [29]. The film thickness was not affected by the addition of the extract.

Moisture content and water solubility are important properties of a biodegradable film that indicate the hydrophilicity of the film and strongly influence its applicability to food [30]. Guar gum is a branched galactomannan polymer consisting of a linear chain of D-mannose units to which D-galactose units are attached laterally, which play a key role in the solubility of the polymer as they interact with water molecules to form hydrogen bonds. The addition of hydrophilic plasticizers such as glycerol and sorbitol can increase the solubility of the film in water as they facilitate the interaction with water molecules and the polymer [11,31,32]. 

Sometimes films require a good moisture barrier and water insolubility for the preservation of sensitive foods; therefore, the addition of a hydrophobic matrix, such as oil, can improve these characteristics to enable different applications. In this case, the medium–low solubility and low moisture content of the films (Table 1) may be effective for application as edible coatings to fresh and minimally processed produce so that food and film are consumed together. Some studies report the incorporation of hydrophobic matrices to guar gum-based films that allow the reduction of moisture content and water solubility, as reported by Aydogdu et al. [31] in other guar gum-based films. Kirtil et al. [30] also report the reduction in moisture content and water solubility of guar gum-based films with glycerol, orange peel oil, and halloysite nanotube. 

Moisture content was not changed by the incorporation of extract from lemon pomace. Similar results were obtained by adding plant extracts to edible films, probably due to the balance between hydrophilic and hydrophilic compounds in the extract [33,34].

The solubility of the film in water decreased with the incorporation of the extract, as also reported in the literature after adding power peel pomegranate on gelatin films [33] and watermelon rind extract to a guar gum film [35]. This could be due to the cross-linking effect of phenolic compounds. The main components of lemon pomace are polyphenols and sugars [36]; the presence of multiple cross-linking agents from simple sugars initiates the formation of covalent bonds, which leads to the reduced water solubility of the films [33]. This could indicate that the addition of the lemon pomace extract (E) was helpful in improving the film’s water resistance.

Edible film produced in this experiment showed low light transmission values, especially in the UV range, indicating a good barrier. Edible films are generally used as surface coatings on products and represent how the product is presented to the consumer; the transparency and opacity of the film are critical parameters that can improve product appearance and consumer acceptance [31,37,38]. The low opacity of G film makes it suitable for applications such as food coating so that high product visibility remains. The color parameters are shown in Table 1; L* values indicate a high film gloss for good visual presentation of the product. In general, the characteristics found are suitable for film application as a surface coating of fresh produce. With the addition of the lemon pomace extract, there is a reduction in film brightness and transparency and an increase in the a* and b* parameters consistent with the visually observed changes related to the yellow-brown color of the extract. The same has been reported in the bibliography with the incorporation of plant extracts to films made with different polymers [39,40]. Results of puncture tests for puncture force showed a similar value for G (1.4 N) and G + E (1.6 N) without significant differences between them and falling within the ranges reported in the bibliography by Rao et al. [37] on chitosan and guar gum films.

**Figure 1:**
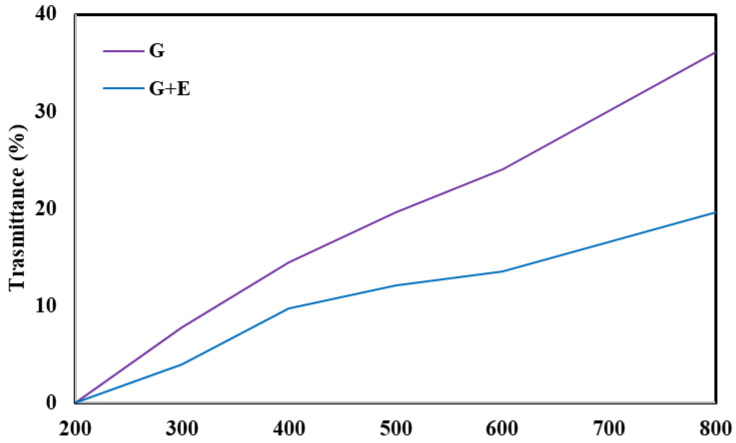
Trasmittance of the films.

G films showed higher light transmittance, probably due to the absence of UV-absorbing groups (Figure 1). With the addition of E, the transmittance of the film decreased due to the presence of absorbing groups such as polyphenols that exhibit strong absorption of UV–Vis radiation and E particles that can block light propagation by dispersing within the matrix. 

Reducing light transmittance can help preserve food because it reduces the influence of light on food. Similar results have been found in the bibliography; in particular, Jiang et al. [39] observed a reduction in luminance in film consisting of chitosan and guar gum after adding walnut green husk extract. In addition, Wang et al. [35] also found the same result after adding watermelon rind extract to a film of guar gum and chitosan.

**Figure 2:**
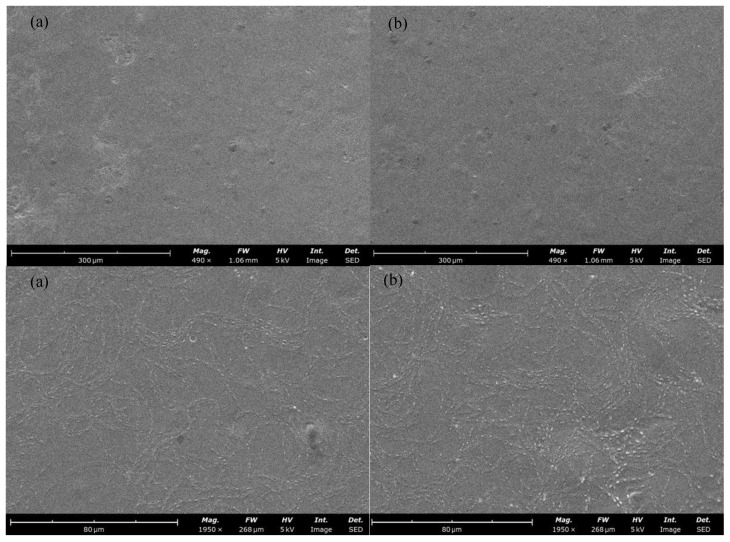
Scanning electron microscopic images of guar gum-based films: (**a**) G, (**b**) G + E (490× and 1950× magnification).

The surface morphology of the G and G + E films observed by scanning electron microscopy is shown in Figure 2. From the images, a continuous structure without cracks is observed in both films indicating that the addition of a low extract concentration did not affect the structure in a major way, as also reported by Wang et al. [35]. It is possible to notice droplets on the surface of both films probably due to the addition of oil in the coating formulation which, being hydrophobic, tends to migrate to the surface. Mutlu [41] reported the observation of the same phenomenon in gelatin/guar gum film incorporated with grape seed oil. 

### 2.2. Characterization of Lemon Pomace Extract (E)

The evaluation of the content of the total polyphenols and flavonoids in the lemon pomace extract allowed us to highlight the consistent content of these compounds, mainly represented by eriocitrin and hesperidin, as confirmed by the literature [27]. The results of antioxidant assays confirmed the effective antioxidant activity provided by the content of phenolic compounds, as already highlighted by Imeneo et al. [28] (Table 2).

### 2.3. Quality Attributes of Cherry Tomato Fruits

#### 2.3.1. Microbiological and Physicochemical Analysis

At the end of storage, the total microbial count showed a significantly lower value (*p* < 0.05) in the coated samples (approximately 2.8 Log CFU g^−1^ in G-B and G-B + E) compared to the uncoated ones (3.0–3.25 log CFU g^−1^ in B and T), as shown in Table 3. Concerning mold counts, highly significant differences were measured at the final time between the samples, with a greater proliferation in the control sample T. The low presence of yeasts at time 0 does not appear to be critical data, also found in Patanè et al. [18], as growth activity ceased during conservation. Contrary to what was reported by Patanè et al. [2], the comparison of T and B shows that the type of packaging influences the level of bacterial proliferation as biodegradable packaging has allowed for levels to be kept lower, with a better effect attributed to the application treatment of the edible coating with guar gum as noted by Ruelas-Chacon et al. [9].

Table 4 shows changes in CIEL*a*b* parameters between samples and over time. The L* values remain relatively stable over time for all treatments, with small variations only for some samples and sometimes reaching final values of around 41–42. Therefore, brightness does not seem to influence the type of preservation packaging or the presence of edible coating. The parameter a* showed a significant increase over time only in T and G-B, indicating intensification of the red color. Also, Ruelas-Chacon et al. [9] observed the increase in red intensity during the storage time of tomatoes, linked to the normal ripening process. The parameter b* showed a slight increase over time in each sample. This result could be associated with an increase in β-carotene, the presence of which influences the yellow/orange color. The H° parameter highlights highly significant differences between the samples, with a considerable reduction over time indicating a tendency towards the red/orange color, except for the G-B + E sample for which the change over time is not significant (*p* < 0.05). The saturation, expressed by the C* parameter, increases slightly over time but only T and G-B showed a considerable variation, with the highest values at the final time. In conclusion, we can therefore make a comparison between our samples: T (stored in PET trays) obtained slightly higher values than B (stored in biodegradable trays) in all colorimetric parameters with significant variation over time.

Consistently with what was stated in Korte et al. [42], the type of packaging has a slight impact on the analysis results. In particular, cellulose trays internally covered with a PLA layer and with PLA lids were used in the study. García-García et al. [43] showed how PLA-coated cardboard trays have better preservation characteristics for tomatoes compared with uncoated trays. The PLA coating absorbs part of the ethylene, delaying its maturation and positively influencing the a/b color parameter. The difference between the behavior of B and that of G-B and G-B + E, all packaged in biodegradable packaging, could be attributed to the guar gum coating, which created a modified atmosphere around the fruits, influencing the rate of respiration and the color change during the storage period itself [9]. The maintenance or slight variation of the color coordinates in B, G-B, and G-B + E, with respect to T, showed a delay of the ripening process, related to the ethylene production and respiration rate in tomatoes, as shown in Zapata et al. [44] on zein-coated tomato fruit.

**Figure 3:**
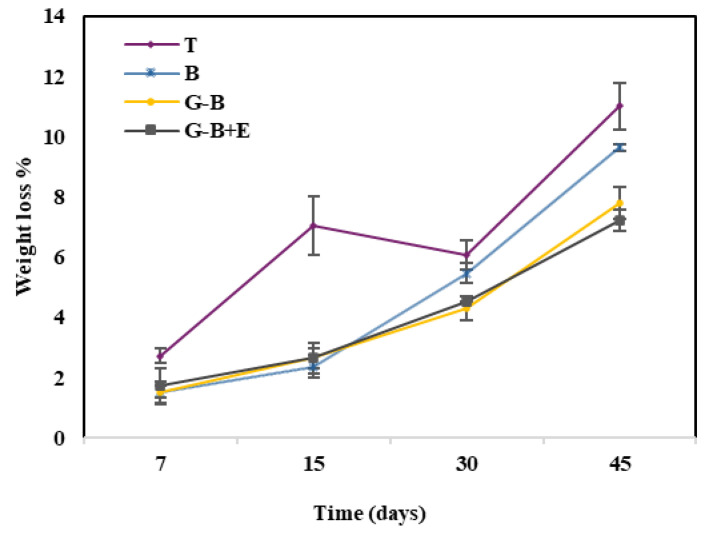
Change in weight loss of cherry tomatoes during storage.

Tomato fruit showed a significant increase (*p* < 0.01) in weight loss in all samples as a function of storage time and treatment (Figure 3). The uncoated samples, stored in conventional and biodegradable packaging, showed, at the final time, the greatest weight loss with values equal to 11.02 ± 0.77% and 9.65 ± 0.1% (Table S1) respectively, highlighting the role of the application of the guar gum coating in the slow weight loss [9]. Furthermore, the incorporation of a hydrophobic part in the coating increases the barrier characteristics by adding to the layer of wax with which the tomatoes are naturally covered, reducing weight loss as found by Adjouman et al. [23] and Yang et al. [45]. Also, Olawuyi et al. [46] found a higher loss of weight due to transpiration phenomena in uncoated tomato than in tomato coated with polysaccharide film composites using mucilaginous polysaccharides and carboxymethylcellulose with extracts from okra leaf waste.

The values obtained for pH, ° Brix, and titratable acidity (TA%) in tomato fruits are represented in Table 5. The analysis of the pH on the tomatoes highlighted increasing values over time in all the samples within a range from 4.1 to 4.4, underlining that the packaging and the use of the coating did not significantly influence the pH of the tomato, as well as also reported by Naeem et al. [1].

Results of total soluble solids (° Brix) highlighted an increase over time, following the normal maturation process which sees the transformation of complex carbohydrates into simple sugars, with values around 5° Brix at 30 and 45 days of conservation. However, uncoated samples showed higher values in a shorter period. The effect of the guar gum coating is probably due to the formation of a semi-permeable barrier around the fruit which modifies the internal atmosphere by slowing down metabolic processes [9].

All samples underwent a significant decrease in titratable acidity (%) over time, starting from a range of approximately 0.5–0.6% citric acid and reaching values of approximately 0.4% citric acid without significant differences between treatments. The decrease in titratable acidity during storage time, both in samples coated with edible and non-edible coatings, was also observed by Ahmed et al. [6] and Shakir et al. [22]. Overall, it was reported that during tomato ripening the ° Brix degree increases and the acidity decreases; this follows the oxidation processes of organic acids and their conversion into sugars [47]. pH, ° Brix, and, titratable acidity values found on tomatoes fall within the ranges reported in coated tomatoes with other natural polymers [48].

The analyses of the lycopene content showed no differences between the various samples (*p* > 0.05) up to time 45, with a slight variation for intermediate times only in B and C-B + E (Table 6). As reported by Azali et al. [49], lycopene variation during storage may be influenced by different factors including packaging, temperature, and oxygen. Furthermore, Barreto et al. [50] also report a decrease in lycopene content in uncoated and coated cherry tomatoes during storage at room or cold temperature.

The analyses of the β-carotene content recorded a significant difference between the control sample, and the other three samples at the end of storage, recording a β-carotene value equal to 83.22 ± 0.94 mg kg^−1^, compared to values ranging from 52.96 ± 6.54 to 69.64 ± 1.99 mg kg^−1^ in B, G-B, and, G-B + E. Furthermore, sample T presented a significant variation over time, ranging from 18.49 ± 3.13 mg kg^−1^ at time 0 to 83.22 ± 0.94 mg kg^−1^ at time 45.

These results agree with Naeem et al. [1], whose study highlighted the possible role of the edible coating made with guar gum in slowing down the synthesis of β-carotene, naturally synthesized during the ripening process of tomatoes through the transition of chloroplasts into chromoplasts where carotenoids are synthesized and accumulated through the conversion of geranylgeranyl pyrophosphate into phytoene and lycopene and the subsequent cyclization of lycopene with the production of α and β carotene [51].

In addition, sample B showed a certain similarity with the coated samples, in particular G-B, and a highly significant difference with the sample packaged in conventional packaging, point to a possible role of biodegradable packaging in slowing down the synthesis processes of β-carotene. This could suggest the important role of packaging in maintaining quality, as confirmed by Azali et al. [49].

#### 2.3.2. Texture Analysis

**Figure 4:**
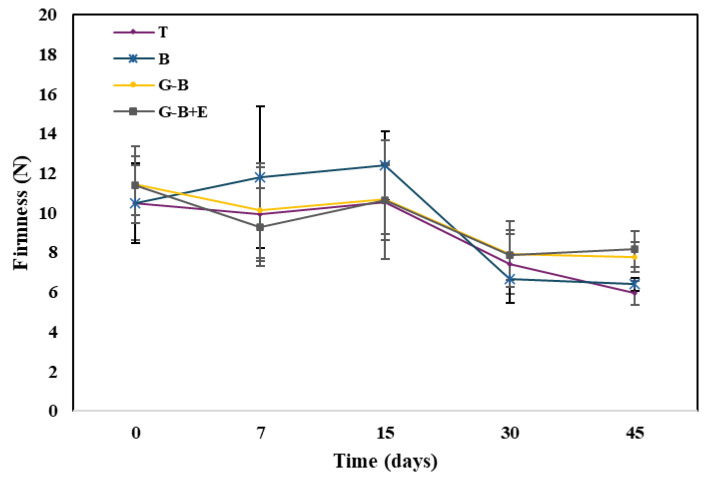
Changes in cherry tomatoes firmness during storage.

The results obtained from the textural analysis are shown in Figure 4, in which it is possible to notice a reduction in the firmness value of approximately 50%, for T and B, compared to the initial time. The smaller reduction is shown in the G-B and G-B + E samples which, characterized by the gel coating, showed higher values at time 45 (7.76 ± 0.76 N and 8.15 ± 0.91 N (Table S2) respectively) compared to the uncoated samples, according to reference [6].

In fact, it has been highlighted that the presence of edible gel coatings in foods preserves the consistency; specifically, the softening of fruits is mainly caused by the deterioration of the cellular structure during the ripening process, due to the action of pectic substances and enzymes that break down the cell wall. The gel coating creates a special atmosphere around the surface of the fruit, increasing CO_2_ levels. As a result, the fruit stays firm longer during storage [48]. Several studies report the application of edible coatings of different origins, such as aloe -based coatings, on tomatoes showing how these have preserved the consistency of fruits over time [52]. In particular, the application of a coating based on antibacterial sulphated rice bran polysaccharides and edible hydroxyethyl cellulose on cherry tomatoes delayed the loss of firmness while maintaining higher values in coated tomatoes than in uncoated ones [53].

#### 2.3.3. Antioxidant Compound of Cherry Tomatoes

The results of the total phenolic content recorded a significant increase (*p* < 0.01) in total polyphenols in all samples during storage (Table 7), as a consequence of ripening, in accordance with what has been reported for different tomato cultivars and different storage conditions [15].

Furthermore, the G-B + E sample recorded, already at time 0, the highest value (292.15 ± 10.83 mg GAE kg^−1^) and maintained a highly significant difference at time 45 (421.17 ± 20.66 mg GAE kg^−1^), compared to the other samples which recorded a total phenolic content within a range from 362.34 ± 6.96 to 388.8 ± 18.28 mg GAE kg^−1^. This would suggest that the lemon pomace extract, used for coating enrichment, influences the phenolic composition of the fruit; this, in fact, increased the phenolic concentration in the tomato, as well as the coating, which probably created an environment around it, with conditions favorable to the synthesis and accumulation of phenolic compounds, as also reported by Kumar et al. [21] and Barreto et al. [50] where the pullulan/chitosan composite coating enriched with pomegranate peel extract enabled the preservation of phenolic compounds during storage at different temperatures.

Moreover, the results obtained are in agreement with what was stated by Shakir et al. [22], whose study highlighted a lower phenolic content in the uncoated samples and an increase in the coated ones, which would suggest an important role on the part of the coatings in maintaining the integrity of the various substrates which normally come into contact during senescence processes and cause oxidation reactions, with a consequent drop in the concentration of phenolic compounds. Furthermore, the application of biodegradable packaging does not seem to particularly influence the concentration of the total phenolic content, as also found in Patanè et al. [18] and Azali et al. [49]. 

The ABTS assay recorded high statistical significance among the samples already at time 0, in which G-B + E showed the highest antioxidant activity with 189.35 ± 5.41 µmol Trolox 100 g^−1^, probably thanks to the lemon pomace extract which provided a content of phenolic compounds, while T and B, both without coating, recorded the lowest antioxidant activity, with 139.00 ± 7.98 µmol Trolox 100 g^−1^ and 145.02 ± 7.83 µmol Trolox 100 g^−1^ respectively. Data confirmed by the determination of the correlation coefficient which, at time 0, highlights a high correlation between the antioxidant assay and the total polyphenol content (r = 0.883).

At time 45, there was a highly significant difference between the samples B, G-B, and G-B + E, and T. The first three samples recorded a similar antioxidant activity, within a range from 193.59 ± 2.75 to 200.98 ± 0.38 µmol Trolox 100 g^−1^, while the lowest antioxidant activity was observed in T, with a value equal to 157.82 ± 2.89 µmol Trolox 100 g^−1^. In this case, the correlation with the total polyphenol content is absent but the antioxidant activity seems to be correlated to the presence of other lipophilic and hydrophilic compounds such as lycopene (r = 0.908) and citric acid (r = 0.933) and not with polyphenols (r = 0.0)

The DPPH assay reported lower antioxidant activity values than the ABTS assay. No relevant differences were observed between the samples at the various monitoring times, except after 45 days in which the greatest antioxidant activity was expressed by the tomatoes coated and packaged in biopackaging. This suggests a possible role of biodegradable packaging in prolonging the shelf life of tomatoes, with consequent maintenance of their antioxidant activity, as also reported in Patanè et al. [18].

The results obtained from the DPPH assay are in agreement with those obtained by Shakir et al. [22], in which the antioxidant activity of the various tomato samples presented very similar values during the first days of storage, and then differentiated significantly thereafter. This could be caused by a slowdown of the ripening processes by the coating on tomato fruits, as also indicated by Tsague Donjio et al. [54]. At the same time, however, sample B also showed the same trends in maintaining its antioxidant capacity, suggesting that this was possible thanks to biopackaging, thus requiring this aspect to be further investigated in future studies.

The role of organic acids, in particular citric and malic acid which represent the main acids present in tomatoes, is important in the quality and organoleptic characteristics of the fruit [5]. The results obtained from the determination of the organic acid content did not show high statistical significance between the treatments for all acids up to time 45, except in citric acid content (Table 8). In fact, a decreasing trend over time was observed with significant differences between samples. Specifically, after 45 days, B, G-B, and G-B + E showed higher contents than T; these values are in line with the normal metabolism of citric acid in tomatoes (tricarboxylic acid cycle), which tends to decrease with the advancement of cellular maturation and respiration processes; however, the higher levels of citric acid recorded on samples with edible coating would confirm the role of the latter in slowing down the aging processes of tomatoes [1].

The determination of the oxalic acid content allowed us to record an increasing trend over time for all samples, but a significant one in B, G-B, and G-B + E; while the malic acid content was stable over time with a significant reduction only in G-B + E.

Generally, ascorbic acid levels can increase with ripening and decrease in the senescence phase [5]; in this case, the ascorbic acid content of the tomato samples was stable for up to 45 days, showing no differences between the various treatments.

However, the organic acid values found fall within the ranges reported in the bibliography [44].

#### 2.3.4. Sensorial Analysis

The results of the sensory analysis at time 0 denote a positive absence of variation as a result of the treatments applied compared to the traditionally packaged tomato that is well accepted by the consumer (Table 9). At time 45, we noted a general decrease in the various descriptors with, in particular, a greater effect found in the control sample T which is the most depreciated in storage, for example, with an overall appearance that from 8.5 ± 0.5 at initial time was judged with 4.4 ± 1.1 at final time. 

Also, for the descriptor “surface uniformity,” sample T (PET packed) turns out to be the worst (4.2 ± 0.8), whereas the better results of B, G-B, and G-B + E could indicate that the presence of the biopackaging and coating is a positive barrier for the preservation of the physical characteristics of the product. Among the coated samples, the crispness of the G-B sample at time 45 was better, while G-B + E scored slightly lower; these results are confirmed by the color and texture analysis.

At the end of the storage, only sample T was not within the limit of 4.5, while sample B showed the best results for general appearance, color, crunchiness descriptors, and total consumer acceptability, followed then by sample G-B and G-B + E, stored in biodegradable packaging; therefore, at the sensory analysis, such packaging gave positive results. In addition, the presence of edible coatings on the samples had no significant negative effects on sensory characteristics, in particular, for the coating enriched with lemon pomace extract, no relevant citrus flavour was perceived.

## 3. Conclusions

The use of a guar gum-based composite gel coating has been shown to be beneficial to the maintenance of the compositional characteristics of the tomatoes, especially when coupled with biodegradable packaging and an enrichment based on lemon pomace extract. The highest phenolic content during tomato storage (292.15 ± 10.83 mg GAE kg^−1^ to 421.17 ± 20.66 mg GAE kg^−1^) found in G-B + E would suggest the active role of the extract obtained from lemon pomace in enriching the coating by positively affecting antioxidant activity (189.35 ± 5.41 µmol Trolox 100 g^−1^ for G-B + E while T and B, both without coating, 139.00 ± 7.98 µmol Trolox 100 g^−1^ and 145.02 ± 7.83 µmol Trolox 100 g^−1^ respectively). The edible gel coating and biodegradable packaging would seem to be able to significantly slow down the microbial growth (approximately 2.8 Log CFU g^−1^ in G-B, and G-B + E, and, 3.0–3.25 log CFU g^−1^ in B and T at the end of storage) and the advancement of ripening processes in tomatoes, with maintenance of texture (5.96 ± 0.61 N, 6.39 ± 0.32 N, 7.76 ± 0.76 N, and 8.15 ± 0.91 N, respectively, in T, B, G-B, and, G-B + E), and weight, as well as with important repercussions on the content of lycopene, β-carotene, and citric acid, which maintains a higher concentration for longer. Edible gel coatings and biodegradable packaging, therefore, stand as a viable alternative to the plastics conventionally in use today, laying the foundation for a future food packaging industry that is more technologically advanced, zero-impact, and capable of positively affecting the maintenance of the quality characteristics of food products.

## 4. Materials and Methods

### 4.1. Experimental Materials

Tomatoes (*Solanum lycopersicum*, L. 1753) were transferred to the Food Technology Laboratory of the Mediterranean University of Reggio Calabria where they were subjected to a sanitization pretreatment in a 200 ppm chlorinated water solution for 2 min and air-dried at room temperature (22 ± 2 °C) under a vertical laminar flow hood (UV lamp 30 W, mod. ASALAIR 1200 FLV, Asal Srl, Milan, Italy) to prevent environmental contamination. Lemon pomace (*Citrus limon* (L.) Osbeck) was supplied by a company located in Reggio Calabria, Italy.

### 4.2. Preparation and Characterization of Antioxidant Extract from Lemon Pomace (E)

The extraction of antioxidant compounds from lemon pomace was carried out mixing 100 g of lemon pomace in 400 mL of ethanol/water (1:1 *v/v*) and keeping the solution under continuous stirring for 30 min at 70 °C [28]. Subsequently, the mixture was centrifuged at 6000 rpm for 10 min (NF 1200R centrifuge refrigerated at 4 °C) and filtered with a Büchner filter and 0.45 μm filter paper. The obtained extract (E) was characterized for the content in polyphenols, flavonoids, and antioxidant activity as reported by Imeneo et al. [28].

### 4.3. Preparation of Edible Gel Coatings

For gel coating (G) formulation, 1% guar gum (GG) (ACEF, Fiorenzuola d’Arda (PC) Italy) was homogenized at 60 °C for 30 min with 20% sorbitol (*w*/*w* GG) (ACEF, Italy) and 20% glycerol (*w*/*w* GG) (Carlo Erba reagents, Italy) as plasticizers, 0.2% (*w*/*v*) extra virgin olive oil and 0.2% Tween 20 (*w*/*v*) as lipid part and emulsifying agent [12]. Another gel coating was formulated by adding 5% (*v*/*v*) of lemon extract (E) to G to obtain G + E sample. The above-mentioned percentage was tested and found to be appropriate as it did not provide negative sensory perception. The prepared solutions were subjected to ultrasonication for degassing treatment and then poured into polystyrene Petri dishes (90 mm diameter) and allowed to dry at 25 °C. The obtained films were stored at 25 °C before analysis.

### 4.4. Characterization of the Developed Edible Film

#### 4.4.1. Thickness

Film thickness was measured using an external precision micrometer (0–25 mm) at 10 random positions for each film, and the average value was calculated. The average value was used in the calculation of optical properties.

#### 4.4.2. Moisture Content (MC) and Water Solubility (WS)

The moisture content of the film was determined by drying at 105 °C until it reached constant weight and calculated as follows:

$$\mathrm{MC}\%=\frac{{(W}_{0}-W_{1})}{W_{0}}\times 100$$

where W_0_ = initial weight of the sample; W_1_ = weight of the sample after drying in the oven calculated [40].

After, the films were immersed in 50 mL of distilled water and placed in constant agitation for 24 h at room temperature to assess water solubility. Then, they were dried again at 105 °C until a constant weight was reached, and the solubility of the film was calculated with the following formula:

$$\mathrm{WS}\;(\%)=\frac{{(M}_{0}-M_{1})}{M_{0}}\times 100$$

where M_0_ is the initial mass after drying of the film and before immersion in water, and M_1_ is the final mass of the film after immersion in water and drying [55].

#### 4.4.3. Optical Properties and Color

The optical properties and color of the film were evaluated as reported by Rao et al. [37] and Nogueira et al. [56]. Film color was measured using a colorimeter (Konica Minolta CM-700d, Inc., Sakai, Osaka, Japan). A white base was used for instrument calibration and as a background for measurements obtained at 10 different points on the film. The CIELab system L* (brightness), a* (red/green color intensity), and b* (yellow/blue color intensity) were used.

A UV–Vis spectrophotometer (Perkin-Elmer UV–Vis 2, Waltham, MA, USA) at wavelengths between 200 and 800 nm was used to evaluate the light transmission of the film. A film sample was placed in a spectrophotometer measurement cell, and air was used as a reference. The average of ten spectra was used to calculate the transparency of the film at 600 nm as

$$T\;(\%)=\log\;\frac{\%T600}{d}$$

where %T600 is the percent transmittance at 600 nm and d is the film thickness (mm). 

Film opacity was calculated as absorbance at 600 nm/film thickness (mm) [31].

#### 4.4.4. Mechanical Properties (Puncture Test)

Determination of the mechanical properties of the film was carried out using a TA-XT Plus Texture Analyzer (Stable Micro Systems Ltd., Godalming, UK) by means of a penetration test with a 5 mm probe, 5 kg load cell, pre-test speed of 1.50 mm/s, test speed of 1 mm/s, post-test speed of 10 mm/s, and trigger speed of 5 g. Measurements were made on three circular film samples (diameter~90 mm) with 2 replicates each [56]. The results were expressed as N (Newton). 

#### 4.4.5. Scanning Electron Microscopy (SEM)

A Scanning Electron Microscopy (Thermoscientific Phenom XL G2 Desktop SEM, Ferentino, Italy) was used to evaluate the morphological characteristics of the surface of the film. The film pieces were mounted on cylindrical aluminum stubs with the aid of double-sided carbon tape and sprayed with a thin layer of gold. After gold coating, the film was observed in SED mode, an acceleration voltage of 5 kV at the desired magnification.

### 4.5. Application of Gel Coatings on Fruits

**Figure 5:**
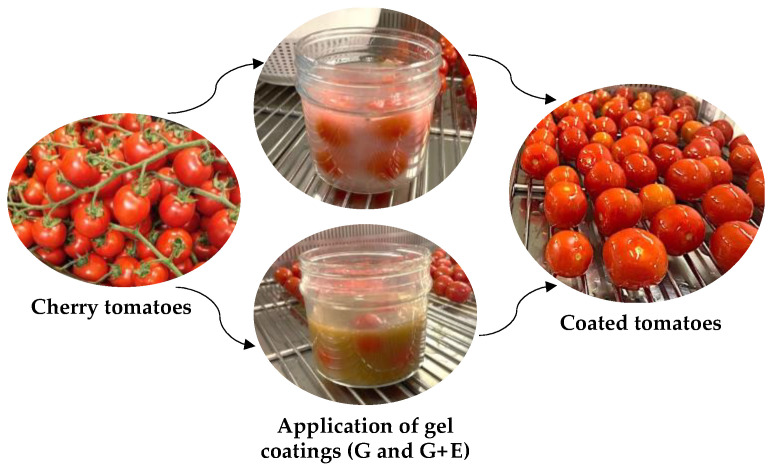
Overview of the preparation process of coated tomatoes.

Immediately after the washing and sanitizing operation, uncoated tomatoes were packaged in bio-based compostable trays (B) and in PET clamshell trays (Imballaggi360 S.r.l., Urbania, Italy, 500 cm^3^ of volume) (T). To produce gel-coated samples, cherry tomatoes were dipped for 30 **s** in G and G + E, and allowed to dry under UV light to prevent any environmental contamination (Figure 5). Coated tomatoes were then packed in bio-based compostable trays (cellulose + PLA lid) (TecnaFood, Bomporto (Modena), Italy, 500 cm^3^ of volume) to obtain G-B and G-B + E samples. All samples were stored at 20 °C and changes in physicochemical, microbiological, and sensory parameters were weekly monitored up to 45 days.

### 4.6. Microbiological and Physicochemical Analysis

For the microbiological analyses, samples were homogenized in a Ringer solution (1:10) for 3 min using a stomacher (BagMixer^®^ 400 P, Interscience, Saint-Nom-la-Bretèche, France). Each sample was serially diluted (1:10) and inoculated, for yeast and mold counts on plates of DRBC Agar, incubating for 4–5 days at 25 ± 2 °C, and for total bacterial counts, on plates of PC Agar, incubating for 48 h at 25 ± 2 °C. The results were reported as Log10 colony-forming units (cfu) g^−1^. 

Surface color was measured using a colorimeter (CM-700d Konica Minolta, Inc., Sakai, Osaka, Japan) at four points of the surface of each tomato. The CIE L*a*b* color space was used as a reference and hue angle (H°) and Chroma (C*) was calculated [36]. 

The weight loss was calculated using a digital balance as the difference between the initial and final weight monitored at the identified storage time. The results were expressed as the percentage of weight loss (%). 

The total soluble solid (TSS) and pH values were determined through the homogenization of 20 g of tomato in an Ultra-Turrax (T 25 digital, IKA, Staufenim Breisgau, Germany). The obtained tomato juice was analyzed with a digital refractometer (ATAGO PR-101) for TSS, with a digital pH meter (pH 4, pH 7; Crison Basic 20, Barcelona, Spain) for the pH according to the AOAC [57]. 

For TA (total acidity %) determination, the diluted juice was titrated against NaOH (0.1 N) until the solution reached a pH of 8.1. The results are expressed as citric acid % values [24]. 

The method reported by Luengo et al. [58], with appropriate modifications, was used to evaluate the β-carotene and lycopene content. A 1 g of sample was mixed with 20 mL of hexane–acetone–methanol (50:25:25 *v*:*v*:*v*) solution. The obtained mixture was homogenized by vortexing for 2 min and the upper hexane phase was removed. The absorbance of the top layer was determined at 663 nm, 645 nm, 505 nm, and 453 nm using UV–Vis spectrophotometer (Perkin-Elmer UV-Vis λ2, Waltham, MA, USA). The contents of lycopene and β-carotene were calculated and expressed as mg kg^−^^1^ [15]:

Lycopene = − 0.0458A_663_ + 0.204A_645_ + 0.372A_505_ − 0.0806A_453_

β-carotene = 0.216A_663_ − 1.22A_645_ − 0.304A_505_+ 0.452A_453_

### 4.7. Extraction and Determination of Antioxidants Compounds

A total of 5 g of tomato pulp was homogenized in 10 mL of methanol solution (80%), centrifuged at 9000 rpm for 10 min at 4 °C. Supernatant was then filtered with a syringe filter (RC, 0.45 μm, diameter 15 mm) and used for the estimation total phenolic content and antioxidant activity [22]. For total phenolic content (TPC) determination, 100 μL of methanolic extract, 1.2 mL Na_2_CO_3_ solution (7.5% *w/v*), and 1.5 mL Folin–Ciocalteau reagent (diluted 1:10 *v/v* with distilled water) were mixed and shaken vigorously. The reaction mixture was incubated in the dark for 90 min at environmental conditions. The absorbance was measured at 750 nm versus a blank (sample replaced by water) using a spectrophotometer (Perkin-Elmer UV–Vis k2, PerkinElmer Inc., Waltham, MA, USA). The total phenolic content was expressed as mg gallic acid equivalent kg^−1^(mg GAE kg^−1^).

Antioxidant assays (DPPH and ABTS) were performed using the method reported by De Bruno et al. [59] with appropriate modifications.

For the DPPH assay, a 6 × 10^−5^ M methanolic solution of 2,2-diphenyl-1-picrylhydrazyl (DPPH) was formulated. The assay was performed by reacting 50 μL of methanolic extract with 2950 μL of methanolic solution of DPPH radicals. The mixture was incubated for 30 min in the dark at room temperature and the absorbance was measured at 515 nm by spectrophotometer (Perkin-Elmer UV–Vis k2, PerkinElmer Inc., Waltham, MA, USA) using methanol as a blank. 

The ABTS assay was performed by reacting a solution of 2,2-azino-bis(3-ethylbenzothiazolin-6-sulfonic acid) with potassium persulfate. Samples were analyzed by mixing 50 μL of ethanolic solution with 2950 μL of ABTS; these were incubated for 6 min in the dark at room temperature. The absorbance was determined spectrophotometrically using ethanol as a blank. The results of the DPPH and ABTS assays were expressed as µmol Trolox 100 g^−^^1^. 

The organic acid content was determined following the method reported by Zapata et al. [44]. The tomato juice was filtered through a 0.45 μm cellulose syringe filter and then injected in an HPLC as reported by De Bruno et al. [59]. A Knauer HPLC Smartline Pump 1000 was used, equipped with a Knauer Smartline 2600 UV detector set at 254 nm for ascorbic acid and 210 nm for oxalic, malic and citric acid, using a Synergi Hydro-RP column (250 mm long × 4.6 mm i.d., 4 μm Phenomenex, Torrance, CA, USA). Chromatographic analysis was conducted using 20 mM of potassium phosphate (pH 2.9) as the mobile phase at a temperature of 22 °C and a flow rate of 0.7 mL/min. External standards as ascorbic acid (Aldrich Chemical Company Inc., Milwaukee, MI, USA), oxalic acid (Carlo Erba, Cornaredo, Italy), malic acid, and citric acid (Sigma Aldrich, Vienna, Austria) were used for quantification of organic acids, and results were expressed as mg 100 g^−^^1^.

### 4.8. Texture Analysis

The texture of cherry tomatoes was determined using a Texture Analyzer (TA-XT Plus, Stable Micro Systems Ltd., Godalming, UK). A cylindrical flat-end probe of 2 mm diameter (P/2) was used for the penetration test, with the following instrumental settings: trigger force of 25 g, pre-test speed of 1.50 mm/s, test speed of 2 mm/s, post-test speed of 10 mm/s, and pierced 10 mm into the tomato. Six replicates were used in each test for all samples. The Exponent software 6.1.4.0 was used for data acquisition and analysis. The peak force (firmness, N) under the force–time curve was obtained.

### 4.9. Sensorial Analysis

Sensory evaluation was conducted at the beginning and after 45 days of storage. Judges (between 23 and 43 years old) with previously experience in sensory analyses assessed samples for visual appearance (general appearance, color, surface uniformity, color saturation), aroma intensity (general flavor, citrus flavor, persistence), taste (sweetness, acidity), and texture (crunchiness, juiciness). On the base of all the sensorial parameters, judges attributed an overall acceptability. Sensory analysis was based on a hedonic scale from 1 to 9 points. A score of 4.5 was considered the limit of acceptability.

### 4.10. Statistical Analysis

All results were statistically processed and expressed as mean value ± SDs. Significant differences (*p* < 0.05) were determined with Turkey’s post-hoc test by one way analysis of variance (ANOVA) performed using SPSS software (version 26.0, SPSS Inc., Chicago, IL, USA). The Pearson’s correlation test was performed to determine correlation coefficients (r) among bioactive compounds and antioxidant assays.

## Figures and Tables

**Table 1:** Physical properties of G and G + E films.

	G	G + E	Sign.
*MC (%)*	17.05 ± 2.94	16.14 ± 0.40	ns
*WS (%)*	40.37 ± 0.9	33.26 ± 3.16	*
L*	88.82 ± 0.98	62.34 ± 3.83	**
a*	−0.51 ± 0.10	11.69 ± 1.88	**
b*	5.96 ± 1.49	33.15 ± 1.57	**
*Transparency*	10.97 ± 0.04	5.98 ± 0.07	**
*Opacity*	9.45 ± 0.19	17.83 ± 0.72	**
*Puncture force (N)*	1.4 ± 0.44	1.6 ± 0.26	ns

Abbreviations: **, significance at *p* < 0.01; *, significance at *p* < 0.05; ns, not significant.

**Table 2:** Results of characterization analysis of the extract from lemon pomace.

E	*TPC (mg GAE mL^−1^)*	7.73 ± 0.09
	*TFC (mg CE mL^−1^)*	3.19 ± 0.0
	*ABTS (mmol TE L^−1^)*	13.19 ± 5.02
	*DPPH (mmol TE L^−1^)*	3.24 ± 0.16
	*Eriocitrin (mg L^−1^)*	879.35 ± 18.98
	*Esperidin (mg L^−1^)*	332.56 ± 21.00

**Table 3:** Microbiological counts of cherry tomatoes (Log10 CFU g^−1^).

	T	B	G-B	G-B + E	*Sign.*
*Total bacterial count (Log10 CFU g^−1^)*					
0	0.00	0.00	0.00	0.00	ns
45	3.25 ± 0.21 ^a^	3.02 ± 0.07 ^ab^	2.87 ± 0.08 ^b^	2.84 ± 0.08 ^b^	*
*Sign.*	**	**	**	**	
*Molds (Log10 CFU g^−1^)*					
0	0.00	0.00	0.00	0.00	ns
45	1.77 ± 0.12 ^a^	0.76 ± 0.00 ^c^	1.44 ± 0.07 ^ab^	1.14 ± 0.12 ^b^	**
*Sign.*	**	**	**	**	
*Yeasts (Log10 CFU g^−1^)*					
0	0.15 ± 0.10	0.15 ± 0.11	0.15 ± 0.12	0.15 ± 0.13	ns
45	0.00	0.00	0.00	0.00	ns
*Sign.*	ns	ns	ns	ns	

Small letters within a row show significant differences as assessed by Tukey’s post hoc test. Abbreviations: **, significance at *p* < 0.01; *, significance at *p* < 0.05; ns, not significant.

**Table 4:** Colorimetric coordinates of cherry tomatoes during storage.

	T	B	G-B	G-B + E	*Sign.*
L*					
0	40.96 ± 0.81 ^abAB^	40.96 ± 0.81 ^ab^	41.67 ± 1.39 ^aAB^	40.89 ± 1.65 ^b^	*
7	40.56 ± 0.85 ^bB^	41.27 ± 0.91 ^a^	41.12 ± 0.64 ^aBC^	41.2 ± 0.64 ^a^	**
15	41.05 ± 0.85 ^AB^	41.12 ± 0.7	40.93 ± 0.75 ^C^	41.14 ± 0.87	ns
30	41.27 ± 0.88 ^A^	41.3 ± 0.96	41.06 ± 0.62 ^BC^	40.92 ± 0.63	ns
45	41.43 ± 0.78 ^abA^	41.09 ± 0.75 ^b^	41.85 ± 0.73 ^aA^	41.26 ± 0.87 ^b^	**
*Sign*.	**	ns	**	ns	
a*					
0	12.84 ± 2.19 ^aCD^	12.84 ± 2.19 ^a^	11.19 ± 2.33 ^bC^	11.43 ± 2.52 ^ab^	**
7	12.16 ± 2.06 ^D^	12.8 ± 2.75	13.64 ± 2.06 ^B^	12.52 ± 1.87	ns
15	13.67 ± 2.28 ^aBC^	13.45 ± 2.44 ^ab^	13.17 ± 2.19 ^abB^	12.22 ± 1.57 ^b^	*
30	14.67 ± 1.71 ^aAB^	13.92 ± 2.07 ^a^	14.37 ± 1.56 ^aB^	12.49 ± 1.77 ^b^	**
45	15.36 ± 2.12 ^abA^	14.30 ± 1.99 ^b^	16.30 ± 1.71 ^aA^	12.76 ± 1.96 ^c^	**
*Sign*.	**	ns	**	ns	
b*					
0	10.8 ± 1.07 ^aAB^	10.8 ± 1.07 ^a^	10.89 ± 2.33 ^aAB^	8.89 ± 2.03 ^bB^	**
7	10.46 ± 0.93 ^abB^	10.9 ± 1.45 ^a^	10.67 ± 1.27 ^abAB^	10.1 ± 0.9 ^bA^	*
15	11 ± 1.64 ^aAB^	10.74 ± 1.41 ^ab^	10.25 ± 1.2 ^abB^	9.93 ± 1.15 ^bAB^	**
30	11.02 ± 1.28 ^aAB^	10.92 ± 1.54 ^a^	10.26 ± 1.18 ^aB^	9.11 ± 1.36 ^bAB^	**
45	11.67 ± 1.60 ^aA^	10.44 ± 1.24 ^bc^	11.46 ± 1.19 ^abA^	9.75 ± 1.52 ^cAB^	**
*Sign*.	*	ns	*	**	
*H°*					
0	40.41 ± 3.73 ^bA^	40.41 ± 3.73 ^bA^	44.31 ± 5.88 ^aA^	37.97 ± 7.88 ^b^	**
7	41.04 ± 3.89 ^aA^	40.8 ± 4.77 ^aA^	38.19 ± 2.24 ^bB^	39.16 ± 2.91 ^ab^	**
15	39.05 ± 2.42 ^AB^	40.02 ± 10.29 ^A^	38.15 ± 3.32 ^B^	39.22 ± 3.82	ns
30	36.89 ± 2.45 ^abC^	38.19 ± 2.53 ^aAB^	35.54 ± 2.03 ^bC^	36.12 ± 2.42 ^b^	**
45	37.27 ± 2.36 ^aBC^	36.26 ± 2.52 ^abB^	35.16 ± 1.71 ^bC^	37.41 ± 2.30 ^a^	**
*Sign*.	**	**	**	ns	
C*					
0	16.81 ± 2.19 ^aBC^	16.81 ± 2.19 ^a^	15.7 ± 2.83 ^abC^	14.59 ± 2.68 ^b^	**
7	16.07 ± 1.99 ^C^	16.87 ± 2.77	17.33 ± 2.32 ^B^	16.1 ± 1.91	ns
15	16.93 ± 4.07 ^BC^	17.21 ± 2.5	16.71 ± 2.3 ^BC^	15.78 ± 1.65	ns
30	18.47 ± 2.01 ^aAB^	17.71 ± 2.45 ^a^	17.67 ± 1.86 ^aB^	15.47 ± 2.14 ^b^	**
45	19.35 ± 2.51 ^abA^	17.73 ± 2.22 ^bc^	19.94 ± 2.00 ^aA^	16.07 ± 2.40 ^c^	**
*Sign*.	**	ns	**	ns	

Small letters within a row and capital letters within a column show significant differences as assessed by Tukey’s post hoc test. Abbreviations: **, significance at *p* < 0.01; *, significance at *p* < 0.05; ns, not significant.

**Table 5:** pH, total soluble solid (° Brix), and titratable acidity (% citric acid) of cherry tomatoes.

*Time*	0	7	15	30	45	*Sign*.
*pH*						
T	4.19 ± 0.01 ^bA^	4.2 ± 0.02 ^b^	4.18 ± 0.06 ^b^	4.37 ± 0.04 ^a^	4.32 ± 0.01 ^abB^	**
B	4.19 ± 0.01 ^bA^	4.2 ± 0.01 ^b^	4.13 ± 0 ^b^	4.34 ± 0.03 ^a^	4.38 ± 0.04 ^aAB^	**
G-B	4.1 ± 0.03 ^bAB^	4.17 ± 0.04 ^b^	4.21 ± 0.04 ^b^	4.4 ± 0.01 ^a^	4.36 ± 0.04 ^aAB^	**
G-B + E	4.11 ± 0.03 ^cB^	4.23 ± 0.01 ^bc^	4.24 ± 0.01 ^b^	4.35 ± 0.06 ^ab^	4.46 ± 0 ^aA^	**
*Sign*.	*	ns	ns	ns	*	
*Total soluble solid (° Brix*)						
T	3.95 ± 0.07 ^bB^	4.95 ± 0.07 ^aA^	4.75 ± 0.01 ^aC^	4.95 ± 0.07 ^a^	5 ± 0 ^a^	**
B	3.95 ± 0.07 ^bB^	4.55 ± 0.07 ^aB^	5.00 ± 0.02 ^aC^	4.50 ± 0.28 ^a^	4.60 ± 0 ^a^	**
G-B	4.7 ± 0.14 ^bA^	4.75 ± 0.07 ^bAB^	4.08 ± 0.07 ^cB^	5.25 ± 0.21 ^a^	5 ± 0 ^ab^	**
G-B + E	4.55 ± 0.07 ^abA^	4.55 ± 0.07 ^abB^	4.08 ± 0 ^bA^	5.15 ± 0.49 ^a^	4.8 ± 0 ^ab^	*
*Sign*.	**	*	**	ns	ns	
*TA (%)*						
T	0.51 ± 0.01 ^aC^	0.45 ± 0.07 ^ab^	0.46 ± 0.01 ^ab^	0.36 ± 0.02 ^b^	0.43 ± 0 A^b^	*
B	0.51 ± 0.01 ^aC^	0.49 ± 0.06 ^a^	0.45 ± 0 ^ab^	0.41 ± 0 ^b^	0.41 ± 0 ^b^	*
G-B	0.62 ± 0.01 ^aA^	0.51 ± 0.04 ^ab^	0.47 ± 0.02 ^b^	0.43 ± 0.05 ^b^	0.39 ± 0.02 ^b^	**
G-B + E	0.57 ± 0 ^aB^	0.52 ± 0.01 ^ab^	0.5 ± 0.05 A^b^	0.36 ± 0.03 ^c^	0.39 ± 0.05 ^bc^	**
*Sign*.	**	ns	ns	ns	ns	

Small letters within a row and capital letters within a column show significant differences as assessed by Tukey’s post hoc test. Abbreviations: **, significance at *p* < 0.01; *, significance at *p* < 0.05; ns, not significant.

**Table 6:** Lycopene and β-carotene content of cherry tomatoes during storage.

*Time*	0	7	15	30	45	*Sign.*
*Lycopene (mg kg^−1^ FW)*						
T	124.82 ± 4.38	128.62 ± 20.61	153.51 ± 6.62	122.57 ± 9.76	112.1 ± 9.98	ns
B	138.44 ± 14.77 ^ab^	130.48 ± 7.93 ^ab^	151.7 ± 0.74 ^a^	106.03 ± 8.96 ^b^	127.18 ± 7.33 ^ab^	*
G-B	141.44 ± 23.37	120.02 ± 23.79	148.98 ± 7.09	147.27 ± 2.23	112.65 ± 2.61	ns
G-B + E	148.07 ± 9.85 ^ab^	111.01 ± 7.74 ^b^	158.94 ± 5.14 ^a^	130.16 ± 20.53 ^ab^	131.08 ± 7.24 ^ab^	*
*Sign*.	ns	ns	ns	ns	ns	
*β-carotene (mg kg^−1^ FW)*						
T	18.49 ± 3.13 ^c^	89.65 ± 2.89 ^a^	41.13 ± 8.09 ^b^	78.25 ± 14.14 ^a^	83.22 ± 0.94 ^aA^	**
B	25.78 ± 1.25	35.42 ± 19.68	59.39 ± 4.83	48.76 ± 3,37	62.75 ± 3.43 ^BC^	*
G-B	36.1 ± 10.81	53.36 ± 18.28	44.9 ± 0.85	46.33 ± 2,94	69.64 ± 1.99 ^AB^	ns
G-B + E	37.54 ± 7.62 ^c^	66.89 ± 3.19 ^ab^	44.5 ± 0.7 ^c^	75.94 ± 5.48 ^a^	52.96 ± 6.54 ^Cbc^	**
*Sign*.	ns	ns	ns	*	**	

Small letters within a row and capital letters within a column show significant differences as assessed by Tukey’s post hoc test. Abbreviations: **, significance at *p* < 0.01; *, significance at *p* < 0.05; ns, not significant.

**Table 7:** Total phenolics content and antioxidant activity of cherry tomatoes.

*Time*	0	7	15	30	45	*Sign.*
*Total phenolics content (mg GAE kg^−1^ FW)*						
T	249.98 ± 14.93 ^cB^	200.26 ± 10.16 ^d^	300.74 ± 8.14 ^bB^	334.48 ± 24.57 ^b^	388.8 ± 18.28 ^aB^	**
B	240.56 ± 13.84 ^bcB^	222.24 ± 4.16 ^c^	293.58 ± 27.66 ^bB^	360.69 ± 27.07 ^a^	362.34 ± 6.96 ^aC^	**
G-B	278.22 ± 9.98 ^cA^	220.25 ± 17.13 ^d^	318.46 ± 23.84 ^bAB^	351.81 ± 16.85 ^ab^	381.87 ± 2.01 ^aB^	**
G-B + E	292.15 ± 10.83 ^cA^	236.18 ± 19.29 ^d^	343.11 ± 12.09 ^bA^	361.64 ± 20.72 ^b^	421.17 ± 20.66 ^aA^	**
*Sign.*	**	ns	*	ns	**	
*ABTS (µmol Trolox 100 g^−1^ FW)*						
T	139.00 ± 7.98 ^abcB^	131.81 ± 10.91 ^bcB^	126.02 ± 9.06 ^cB^	164.4 ± 9.55 ^a^	157.82 ± 2.89 ^abB^	*
B	136.82 ± 7.83 ^bB^	129.75 ± 1.63 ^bB^	117.52 ± 0.42 ^bB^	149.52 ± 20.78 ^b^	193.59 ± 2.75 ^aA^	**
G-B	148.43 ± 9.35 ^AB^	147.08 ± 5.05 ^AB^	149.65 ± 6.43 ^A^	154.52 ± 4.61	200.98 ± 0.38 ^A^	ns
G-B + E	189.35 ± 5.41 ^abA^	164.19 ± 1.19 ^abA^	160.01 ± 2.29 ^abA^	150.93 ± 25.27 ^a^	198.99 ± 3.84 ^aA^	*
*Sign*.	**	*	**	ns	**	
*DPPH (µmol Trolox 100 g^−1^ FW)*						
T	53.5 ± 4.28 ^ab^	47.09 ± 1.56 ^bc^	49.24 ± 0.66 ^bc^	60.69 ± 1.75 ^a^	41.68 ± 2.37 ^cB^	**
B	52.3 ± 3.97 ^ab^	42.98 ± 4.39 ^b^	44.9 ± 5 ^ab^	57.61 ± 4.63 ^a^	53.18 ± 2.2 ^abA^	*
G-B	53.99 ± 3.98	50.12 ± 3.52	49.71 ± 5.43	59.86 ± 1.88	58.2 ± 3.27 ^A^	ns
G-B + E	56.73 ± 1.78	54.12 ± 2.45	54.92 ± 2.97	55.29 ± 11.99	54.27 ± 1 ^A^	ns
*Sign*.	ns	ns	ns	ns	**	

Small letters within a row and capital letters within a column show significant differences as assessed by Tukey’s post hoc test. Abbreviations: **, significance at *p* < 0.01; *, significance at *p* < 0.05; ns, not significant.

**Table 8:** Organic acid content of cherry tomatoes.

*Time*	0	7	15	30	45	*Sign.*
*Citric acid (mg 100 g^−1^ FW)*						
T	419.57 ± 14.58 ^a^	357.35 ± 3.91 ^b^	397.58 ± 7.5 ^aA^	306.26 ± 1.82 ^cBC^	75.33 ± 3.48 ^dB^	**
B	398.06 ± 14.58 ^a^	379.93 ± 11.33 ^ab^	355.87 ± 1.18 ^bcC^	329.95 ± 0.36 ^cAB^	100.36 ± 3.06 ^dA^	**
G-B	432.21 ± 15.63 ^a^	396.58 ± 12.73 ^ab^	382.66 ± 0.35 ^bcAB^	352.18 ± 11.05 ^cA^	114.24 ± 7.04 ^dA^	**
G-B + E	420.83 ± 8.03 ^a^	383.23 ± 19.01 ^b^	371.39 ± 1.58 ^bBC^	294.33 ± 3.75 ^cC^	98.73 ± 1.68 ^dA^	**
*Sign*.	ns	ns	**	**	**	
*Ascorbic acid (mg 100 g^−1^ FW)*						
T	20.45 ± 0.43	16.76 ± 2.2	18.7 ± 1.05	18.82 ± 0.73	19.53 ± 0 ^A^	ns
B	20.63 ± 0.33 ^a^	19.78 ± 1.37 ^ab^	18.7 ± 0.07 ^ab^	15.99 ± 1.78 ^b^	17.6 ± 0.41 ^abAB^	*
G-B	19.32 ± 0.48	22.41 ± 0.31	18.13 ± 3.41	18.5 ± 3.17	20.51 ± 3.56 ^A^	ns
G-B + E	19.14 ± 0.22	21.14 ± 2.13	17.13 ± 0.27	15.26 ± 6.44	10.51 ± 0.11 ^B^	ns
*Sign*.	ns	ns	ns	ns	*	
*Malic acid (mg 100 g^−1^ FW)*						
T	104.59 ± 18.49	87.35 ± 15.17	85.68 ± 2.3	92.77 ± 4.39	97.98 ± 1.96 ^AB^	ns
B	103.86 ± 19.52	85.58 ± 5.05	82.73 ± 3.39	89.13 ± 9.19	83.21 ± 9.65 ^B^	ns
G-B	111.3 ± 1.39	103.96 ± 13.88	96.95 ± 20.98	95.69 ± 8.63	107.28 ± 0.62 ^A^	ns
G-B + E	106.85 ± 1.50 ^a^	91.86 ± 1.05 ^b^	97.36 ± 7.09 ^ab^	95.9 ± 4.37 ^b^	83.14 ± 0.02 ^bB^	**
*Sign*.	ns	ns	ns	ns	*	
*Oxalic acid (mg 100 g^−1^ FW)*						
T	79.32 ± 2.09	92.83 ± 10.65	107.48 ± 6.11	103.6 ± 12.95	104 ± 2.28 ^AB^	ns
B	78.06 ± 4.28 ^b^	86.84 ± 8.69 ^ab^	96.21 ± 2.68 ^ab^	110.02 ± 8.7 ^a^	107.92 ± 2.98 ^aAB^	*
G-B	75.38 ± 4.04 ^b^	109.94 ± 0.05 ^a^	97.2 ± 5.57 ^ab^	104.99 ± 14.42 ^ab^	110.86 ± 5.09 ^aA^	*
G-B + E	76.04 ± 4.81 ^c^	95.2 ± 3.71 ^b^	94.13 ± 1.56 ^b^	116.27 ± 1.06 ^a^	96.58 ± 1.85 ^bB^	**
*Sign*.	ns	ns	ns	ns	*	

Small letters within a row and capital letters within a column show significant differences as assessed by Tukey’s post hoc test. Abbreviations: **, significance at *p* < 0.01; *, significance at *p* < 0.05; ns, not significant.

**Table 9:** Sensorial parameters of cherry tomatoes.

*Time*		0	45	*Sign*.
*General appearance*	T	8.5 ±0.5	4.4 ± 1.1 ^c^	**
	B	8.5 ± 0.5	7.8 ± 0.8 ^a^	ns
	G-B	8.3 ± 0.5	6.8 ± 0.8 ^ab^	*
	G-B + E	8.2 ± 0.8	5.6 ± 0.9 ^bc^	**
*Sign.*		ns	**	
*Color*	T	8.5 ± 0.8	4.8 ± 1.1 ^b^	**
	B	8.5 ± 0.8	7.6 ± 0.5 ^a^	ns
	G-B	7.7 ± 1.0	6.2 ± 1.1 ^ab^	*
	G-B + E	8 ± 0.9	5.2 ± 1.3 ^b^	**
*Sign.*		ns	**	
*Saturation*	T	8.5 ± 0.5	5 ± 1.0 ^b^	**
	B	8.5 ± 0.5	7.2 ± 1.1 ^a^	*
	G-B	7.8 ± 0.8	6.4 ± 0.5 ^ab^	**
	G-B + E	8.2 ± 0.8	5.8 ± 0.8 ^ab^	**
*Sign.*		ns	**	
*Surface uniformity*	T	8 ± 0.9	4.2 ± 0.8 ^c^	**
	B	8 ± 0.9	7.6 ± 1.1 ^a^	ns
	G-B	8.2 ± 0.8	6.4 ± 0.9 ^ab^	**
	G-B + E	7 ± 1.1	5.4 ± 0.9 ^bc^	*
*Sign.*		ns	**	
*General flavour*	T	8 ± 0.6	4.6 ± 0.9 ^b^	**
	B	8 ± 0.6	6.8 ± 0.8 ^a^	*
	G-B	7.7 ± 1.0	4 ± 0.7 ^b^	**
	G-B + E	7.8 ± 0.8	3.8 ± 0.8 ^b^	**
*Sign.*		ns	**	
*Citrus flavour*	T	7.33 ± 1.37	3.40 ± 1.14	**
	B	7.33 ± 1.37	4.80 ± 1.79	**
	G-B	7.17 ± 1.17	3.00 ± 1.58	**
	G-B + E	6.83 ± 1.33	3.00 ± 0.71	**
*Sign.*		ns	ns	
*Crunchiness*	T	6.7 ± 2.3	4.4 ± 1.1 ^b^	*
	B	7.2 ± 1.5	6.4 ± 0.5 ^a^	ns
	G-B	6.7 ± 2.0	6.4 ± 0.5 ^a^	ns
	G-B + E	6.5 ± 1.0	5.2 ± 0.4 ^ab^	*
*Sign.*		ns	**	
*Juiciness*	T	7.8 ± 1.3	2 ± 0	**
	B	7.8 ± 1.3	2.8 ± 0.8	**
	G-B	7.5 ± 1.0	2.6 ± 0.5	**
	G-B + E	7.8 ± 1.0	2.2 ± 0.4	**
*Sign.*		ns	ns	
*Overall acceptability*	T	8.17 ± 0.75	4 ± 1	**
	B	8.33 ± 0.52	7 ± 0.71	**
	G-B	7.83 ± 0.75	5.6 ± 1.34	**
	G-B + E	8.17 ± 0.75	5.8 ± 0.84	**
*Sign.*		ns	ns	

Small letters within a row show significant differences as assessed by Tukey’s post hoc test. Abbreviations: **, significance at *p* < 0.01; *, significance at *p* < 0.05; ns, not significant.

## Data Availability

The original contributions presented in the study are included in the article/Supplementary Material, further inquiries can be directed to the corresponding author/s.

## Supplementary Materials

The following supporting information can be downloaded at: https://www.mdpi.com/article/10.3390/gels10090549/s1, Table S1: Change in weight loss of cherry tomatoes during storage; Table S2: Changes in cherry tomato firmness during storage.
