# Supplementary material for: Impact of Antioxidant-Enriched Edible Gel Coatings and Bio-Based Packaging on Cherry Tomato Preservation
Source: Gels. 2024 Aug 24;10(9):549. doi: 10.3390/gels10090549 (PMC11431231; doi:10.3390/gels10090549)
Supplement: Supplementary file 1 [file gels-10-00549-s001.zip › gels-3158085-supplementary.pdf]

S1. Change in weight loss of cherry tomatoes during storage

	Weight loss (%)				
Time	*7*	*15*	*30*	*45*	*Sign.*
T	2.73 ± 0.25^{c}	7.06 ± 0.99^{bA}	6.07 ± 0.48^{bA}	11.02 ± 0.77^{aA}	**
B	1.51 ± 0.36^{c}	2.38 ± 0.38^{cB}	5.48 ± 0.32^{bAB}	9.65 ± 0.1^{aAB}	**
G-B	1.52 ± 0.2^{c}	2.65 ± 0.5^{bcB}	4.33 ± 0.4^{bB}	7.81 ± 0.53^{aBC}	**
G-B+E	1.73 ± 0.58^{c}	2.66 ± 0.34^{cB}	4.52 ± 0.03^{bB}	7.24 ± 0.36^{aC}	**
Sign.	ns	**	*	**	

Small letters within a row and capital letters within a column show significant differences as assessed by Tukey's post hoc test. Abbreviations: **, significance at $p < 0.01$; *, significance at $p < 0.05$; ns, not significant.

S2. Changes in cherry tomatoes firmness during storage

	Firmness (N)					
Time	0	7	15	30	45	*Sign.*
T	10.51 ± 1.89^{a}	9.93 ± 2.38^{a}	10.50 ± 1.90^{a}	7.41 ± 1.51^{b}	5.96 ± 0.61^{bB}	**
B	10.50 ± 2.02^{a}	11.78 ± 3.57^{a}	12.37 ± 1.72^{a}	6.68 ± 1.23^{b}	6.39 ± 0.32^{bB}	**
G-B	11.43 ± 1.94^{a}	10.11 ± 2.40^{ab}	10.70 ± 1.78^{a}	7.93 ± 1.67^{bc}	7.76 ± 0.76^{cA}	**
G-B+E	11.38 ± 1.49^{a}	9.28 ± 1.97^{abc}	10.64 ± 2.99^{ab}	7.87 ± 1.27^{c}	8.15 ± 0.91^{bcA}	**
Sign.	ns	ns	ns	ns	**	

Small letters within a row and capital letters within a column show significant differences as assessed by Tukey's post hoc test. Abbreviations: **, significance at $p < 0.01$; ns, not significant.
